# Prostaglandin E_2 _regulates the expression of connective tissue growth factor (CTGF/CCN2) in human osteoarthritic chondrocytes via the EP4 receptor

**DOI:** 10.1186/1756-0500-3-5

**Published:** 2010-01-15

**Authors:** Kayo Masuko, Minako Murata, Kazuo Yudoh, Hiroyuki Shimizu, Moroe Beppu, Hiroshi Nakamura, Tomohiro Kato

**Affiliations:** 1 Department of Biochemistry, St. Marianna University School of Medicine, 2-16-1 Sugao, Miyamae-ku, Kawasaki-shi, Kanagawa 216-8511, Japan; 2 Department of Frontier Medicine, Institute of Medical Science, 2-16-1 Sugao, Miyamae-ku, Kawasaki-shi, Kanagawa 216-8512, Japan; 3 Department of Orthopedic Surgery, St. Marianna University School of Medicine, 2-16-1 Sugao, Miyamae-ku, Kawasaki-shi, Kanagawa 216-8511, Japan; 4 Department of Rheumatology, Nippon Medical School, 1-1-5 Sendagi, Bunkyo-ku 113-8603, Tokyo, Japan

## Abstract

**Background:**

The regulatory mechanisms of the expression of connective tissue growth factor/CCN family member 2 (CTGF/CCN2) in human articular chondrocytes have not been clarified. We investigated the effect of prostaglandin E_2 _(PGE_2_) on CTGF/CCN2 expression in chondrocytes.

**Findings:**

Articular cartilage samples were obtained from patients with osteoarthritis (OA) and chondrocytes were isolated and cultured in vitro. Chondrocytes were stimulated with PGE_2_, PGE receptor (EP)-specific agonists, or interleukin (IL)-1. CTGF expression was analyzed using quantitative polymerase chain reaction, Western blot, and enzyme-linked immunosorbent assay. The inhibitory effects of EP receptor antagonists (for EP2 and EP4) against PGE_2 _stimulation were also investigated. Stimulation of chondrocytes with PGE_2 _or IL-1 significantly suppressed CTGF expression. The suppressive effect of PGE_2 _was reproduced by EP2/EP4 receptor agonists but not by EP1/EP3 receptor agonists, and was partially blocked by an EP4 receptor antagonist, suggesting that the EP4 receptor has a dominant role.

**Conclusions:**

PGE_2 _may be involved in the regulation of CTGF/CCN2 expression in human articular chondrocytes via the EP4 receptor. Elucidation of EP4-mediated signaling in chondrocytes may contribute to a better understanding of the effects of PGE_2 _in arthritis.

## Background

Connective tissue growth factor/CCN family member 2 (CTGF/CCN2) is a member of the CCN family, which is a group of secreted multifunctional proteins that contain high levels of cysteine (reviewed in [1]). During skeletal development, CTGF is strongly expressed in the mesenchyme, including in hypertrophic chondrocytes, and plays an essential role in endochondral ossification by promoting angiogenesis, proliferation, and differentiation of chondrocytes [2]. In adult tissue, CTGF is expressed during wound healing and in fibrotic tissue [2,3]. Transforming growth factor (TGF)-β stimulates CTGF expression, and TGF-β-induced CTGF is involved in scarring and fibrogenesis [3]. In chondrocytes, CTGF contributes to the production of the extracellular matrix by stimulating chondrocyte proliferation, the expression of type II collagen, and aggrecan among other factors, and the activation of integrin signaling [4,5]. CTGF expression is detected in normal human cartilage and in cartilage from patients with osteoarthritis (OA), suggesting that CTGF expression is involved in the development of fibrous tissue over the damaged OA cartilage [6,7].

Despite its potential involvement in OA, the regulatory mechanisms of CTGF expression in chondrocytes have not been fully clarified. Studies using chondrocytic cells and other cell types indicate that CTGF expression is modulated by several factors, including TGF-β [8], dexamethasone[9], and macrophage colony stimulating factor [10]. A regulatory role of prostaglandin E_2 _(PGE_2_) in CTGF expression has also been postulated [11,12]. PGE_2 _is considered an important catabolic factor in OA cartilage [13]; therefore, the present study aimed to evaluate the potential contribution of PGE_2 _in the regulation of CTGF expression in OA chondrocytes.

## Methods

### Samples

Human articular chondrocytes were obtained from 17 patients (M/F = 2:15, mean age 74.2 ± 5.38) with OA who underwent arthroplasty of a knee (15) or hip (2) joint at St. Marianna University School of Medicine Hospital. The diagnoses of OA was made according to the criteria of the American College of Rheumatology [14]. Written informed consent was obtained from each patient, and the study protocol was approved by the institution's ethics committee. The study was performed in compliance with the tenets of the Declaration of Helsinki proposed by the World Medical Association in 1964.

### Cell culture

After careful removal of the synovial tissue, the cartilage was minced, washed, and treated with collagenase. Isolated chondrocytes were then washed and cultured in vitro as a monolayer in Dulbecco's modified Eagle's medium supplemented with 10% fetal bovine serum and antibiotics. The fetal bovine serum used in the study was inactivated by incubation at 56°C for 30 min. The attached cells (P0) were grown on type I collagen-coated culture dishes, and subconfluent cells (P1 cells) were used in the experiments. The differentiated phenotypes of the cells used in the experiments were confirmed through macroscopic observation and on the basis of the expression of type II collagen and aggrecan mRNA by reverse transcription-polymerase chain reaction (PCR; data not shown).

Chondrocytes were serum-starved in medium with 0.5% fetal bovine serum for 24 h prior to the experiments and were either stimulated or not stimulated with PGE_2 _(10 nM; Cayman Chemical Co., Ann Arbor, MI), butaprost (10 μM, Sigma-Aldrich, St. Louis, MO), PGE_1 _alcohol (10 nM), sulprostone (1 μM, Sigma-Aldrich), or IL-1β (10 ng/ml, R&D Systems, Minneapolis, MN), for the indicated periods. In a separate experiment, cells were pretreated with either AH6809 (10 ng/ml) or GW627368X (5 μM; Cayman Chemical Co., Ann Arbor, MI) for 1 h before stimulation with PGE_2_. Cell viability was not affected by up to 500 nM PGE_2_, the vehicle, or any of the inhibitors during the culture period, as confirmed by trypan blue exclusion and an MTS [3-(4,5-dimethylthiazol-2-yl)-5-(3-carboxymethoxyphenyl)-2-(4-sulfophenyl)- 2H-tetrazolium] assay (data not shown). Stimulated chondrocytes and culture supernatants were collected and subjected to the following analyses.

### Real-time PCR

Messenger RNA was extracted from the cultured cells and converted to cDNA. Quantitative PCR was performed using specific primers and an ABP Prism 7000 according to the manufacturer's protocol. The sequences for the CTGF primer (GenBank No. NM_001901) were as follows: forward, 5'-CCTGTGCAGCATGGACGTT-3'; reverse, 5'-GGACCAGGCAGTTGGCTCTAA-3', and the sequences for the GAPDH primer were as published elsewhere [15]. The oligonucleotides were synthesized by Takara Bio Co. Ltd. (Japan)

### Western blotting

Whole cell lysates were extracted from the cultured cells using standard lysis buffer (20 mM Tris-HCl, 250 mM NaCl, 1% NP-40, 1 mM dithiothreitol, 10 mM NaF, 2 mM Na_3_VO_4_, 10 mM Na_4_P_2_O_7_), and Protease Inhibitor Cocktail (Roche, Mannheim, Germany) and stored at -30°C until use. Protein concentration was determined using the Bradford method (Bio-Rad Protein Assay Reagent; BioRad Laboratories, Hercules, CA). The lysates were mixed with the loading dye and subjected to 15% sodium dodecyl sulphate (SDS)-polyacrylamide gel electrophoresis (PAGE). After transfer to a polyvinylidine difluoride membrane, a primary antibody was added: goat anti-CTGF polyclonal antibody (Catalog No. sc-14939, Santa Cruz Biotechnology Inc. Santa Cruz, CA, USA) or anti-glyceraldehyde-3-phosphate dehydrogenase (GAPDH; Abcam Ltd., Cambridge, UK). The membrane was then washed and reacted with the corresponding secondary antibody conjugated with horseradish peroxidase. Finally, the signals were visualized using the extended cavity laser system (GE Healthcare Bio-sciences KK, Tokyo, Japan). Densitometry of the signal bands was analysed using ImageJ software http://rsb.info.nih.gov/ij/. Statistical analyses were performed using Prism™ software (GraphPad Software Inc., San Diego, CA). The results are shown as mean ± SD. Student's t test was used to compare between two groups and a *p *value of less than 0.05 was considered significant.

### Enzyme-linked immunosorbent assay (ELISA)

CTGF levels in the culture supernatant were measured in duplicate using the Human CTGF ELISA Kit (Peprotech, Rocky Hill, NJ) according to the manufacturer's instructions. According to the manufacturer, the detection range of CTGF using this kit is 63 to 4000 pg/ml. The results were analyzed using Student's t-test.

## Results

### PGE_2 _downregulates CTGF expression in chondrocytes

**Figure 1:**
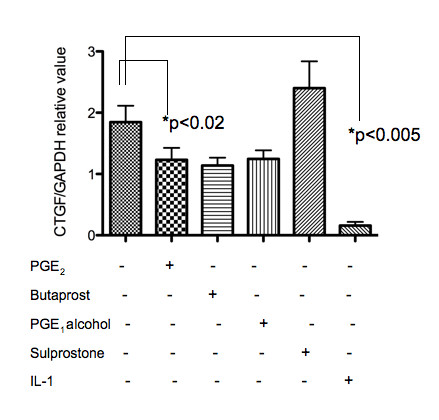
**Quantitative PCR for CTGF expression in PGE_2_- or EP receptor agonist-stimulated human chondrocytes**. Chondrocyte samples (n = 5, in duplicate) were either stimulated or not stimulated with PGE_2 _(10 nM), butaprost (10 μM), PGE_1 _alcohol (10 nM), sulprostone (1 μM), or IL-1β (10 ng/ml) for 24 h in vitro. The relative expression of CTGF mRNA was calculated against values of the internal control GAPDH.

**Figure 2:**
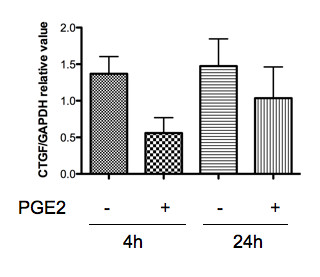
**Quantitative PCR for CTGF expression in chondrocytes: different time points**. Chondrocyte samples (n = 3, a different panel from Figure 1) were stimulated with PGE_2 _(10 nM) for 4 or 24 h, and each sample was analysed for the CTGF mRNA as in Fig. 1.

Figure 1 shows the results of quantitative PCR. IL-1β and, to a lesser extent, PGE_2 _significantly suppressed the expression of CTGF mRNA. We observed a similar effect of PGE_2 _in chondrocyte samples from patients with traumatic fracture and rheumatoid arthritis (data not shown). The suppressive effect of PGE_2 _on CTGF mRNA expression tended to be more potent 4 h after stimulation than at 24 h after stimulation (Figure 2), suggesting that suppression occurred at the transcriptional level. A comparison of the effect of EP agonists and the effect of PGE_2 _indicated that the effects of butaprost (EP2 agonist) and PGE_1 _alcohol (EP2/EP4 agonist) were similar to those of PGE_2_. Sulprostone (EP1/EP3 agonist), however, did not have a suppressive effect. These findings suggest that EP2 and/or EP4 receptors are involved in the suppression of CTGF expression by PGE_2 _(Figures 1). Based on previous reports that butaprost might exert agonistic effects on EP4 at micromolar concentrations in some cells [16], we further analysed the effect of PGE_2 _using EP2/EP4-specific antagonists. An EP4-specific agonist was not commercially available.

**Figure 3:**
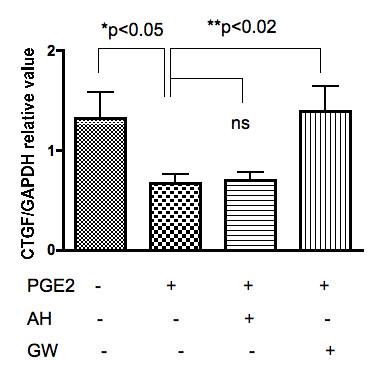
**CTGF mRNA expression in PGE_2_-stimulated chondrocytes: blocking experiments**. Chondrocyte samples (n = 5, in duplicate) were either stimulated by PGE_2 _(10 nM) or not for 24 h, with or without pretreatment with an EP-specific antagonist (AH: AH6809, 10 ng/ml; GW: GW627368X, 5 μM), for 1 h in vitro. The relative expression of CTGF/CCN2 mRNA was calculated against values of the internal control, GAPDH using quantitative PCR.

**Figure 4:**
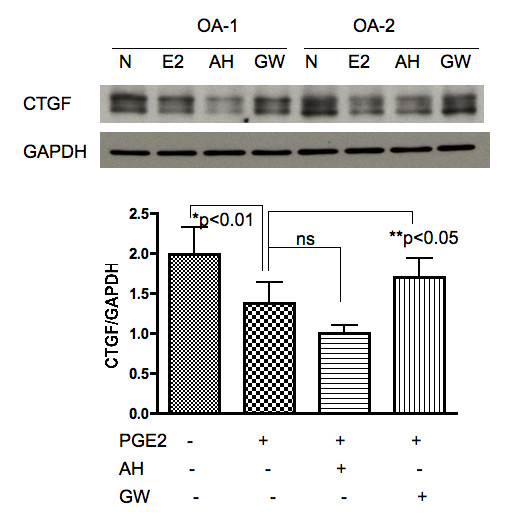
**CTGF expression in PGE_2_-stimulated chondrocytes: Western blot**. Top: Chondrocyte samples (n = 5) were either stimulated by PGE_2 _(10 nM) or not for 24 h, with or without pretreatment with an EP-specific antagonist (AH: AH6809, 10 ng/ml; GW: GW627368X, 5 μM), for 1 h in vitro. Bottom: The relative value of band intensity (CTGF/GAPDH) was calculated using an image analyzer.

Chondrocytes were stimulated with PGE_2 _in the presence or absence of AH6809 (EP2 antagonist) or GW627368X (EP4 antagonist). The results of quantitative PCR using these antagonists revealed that GW627368X, but not AH6809, blocked the suppressive effect of PGE_2 _on CTGF expression (Figure 3), suggesting that the EP4 receptor has a dominant role in the effects of PGE_2 _on CTGF. Western blot analyses produced similar results (Figure 4), confirming that the effects of PGE_2 _were mediated through the EP4 receptor at the protein level. Of note, in the Western blot, CTGF protein was detected as several bands of approximately 30 and 35 kD (Figure 4, top). This finding suggests the presence of cleaved fragments, as reported in previous studies [5,8,17].

### Suppression of CTGF secretion by PGE_2_

**Figure 5:**
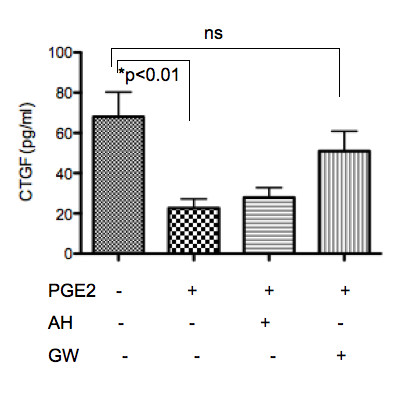
**Secreted levels of CTGF from chondrocytes**. The results of ELISA assays using culture supernatants from OA chondrocytes (n = 5) are summarized. All of the assays were performed in duplicate.

We measured secreted levels of CTGF from cultured OA chondrocytes byELISA. Basal CTGF levels were detected in the culture supernatant of three of five OA chondrocyte samples. In all tested samples, adding PGE_2 _to the cells significantly decreased CTGF secretion (Figure 5). As a result, preincubation with AH6809 did not significantly prevent the decrease in CTGF secretion, whereas GW267368X abolished the suppressive effect of PGE_2 _(Figure 5).

## Discussion and Conclusions

CTGF/CCN2 is currently attracting attention for its possible contribution to chondrocyte metabolism, including chondrogenesis, chondrocyte proliferation, and extracellular matrix production [4]. Although CTGF is expressed in human OA chondrocytes as well as in normal human chondrocytes [6,7], the regulatory mechanisms of its expression are not fully understood. Here, we evaluated whether the catabolic mediator PGE_2 _is involved in the regulation of CTGF expression in articular chondrocytes. Both PGE_2 _and IL-1β suppressed CTGF expression in human articular chondrocytes, and the inhibitory effect of PGE_2 _was EP4 receptor-dependent.

PGE_2 _exerts its effects through the family of EP receptors, i.e., EP1, EP2, EP3, and EP4. Each EP receptor has a distinct distribution pattern and shows different responses after PGE_2 _stimulation (reviewed in [18]). EP2 and EP4 receptors are suggested to deliver important signals to chondrocytes [19-21]. In this regard, Aoyama et al. previously reported the dominant expression of EP2 receptors in human articular cartilage and cultured chondrocytes, whereas significant expression of EP1 and EP4 receptors was not detected [20]. Otsuka and Aoyama et al. also reported that selective stimulation of the EP2 receptor promoted cartilage regeneration in an animal model with chondral defects [22], suggesting that EP2 has a dominant role in chondrocyte proliferation and cartilage regeneration. Attur et al. [13], however, recently demonstrated that all four EP receptors (EP1-EP4) are expressed in both OA and normal cartilage and in chondrocytes, and that the catabolic effects of PGE_2 _are mediated by signaling through EP4 receptors in OA cartilage. Specifically, the authors demonstrated that PGE_2_-induced matrix metalloproteinase production and type II collagen degradation were both EP4-dependent [13]. In addition, Li et al. demonstrated the predominant expression of EP2 and EP4 receptors in human articular cartilage, and showed that PGE_2 _works as a potent anti-anabolic factor via the suppression of proteoglycan production, which suggests a potential therapeutic application of EP2 and EP4 receptor antagonists [21]. Our results are consistent with their findings in regard to the implication of the involvement of EP4 receptors in the catabolic response to PGE_2 _in chondrocytes. Specifically, CTGF mRNA and protein expression was suppressed by an EP4-selective antagonist. Thus, regulation of the EP4-mediated signal might be an important target to suppress the degradation process of inflamedarticular cartilage. Of note, Li et al. also reported that the expression levels of each EP receptor might vary between knee and ankle joints [21]. In the present study, we used 15 knee samples and 2 hip joints without any significant difference in the CTGF responses; however, further studies should be performed to clarify whether chondrocytes in various joints respond differently to PGE_2_.

CTGF has several potential functions in chondrocytes. During chondrogenesis, CTGF has an important role as a regulator of matrix remodeling and chondrocyte differentiation [3,23,24]. Woods et al. recently demonstrated that CTGF expression in chondrocytes is regulated by a small GTPase Rac1 and actin organization via the Smad binding site in the *CTGF *promotor gene, suggesting the involvement of TGF-β/smad signaling in CTGF expression [25]. On the other hand, using rabbit auricular (not articular) cartilage, Fujisawa et al. reported that CTGF might increase proteoglycan synthesis and the expression of elastin and type II collagen, whereas it suppresses apoptotic cell death [26]. Another recent paper by Maeda et al. reported that CTGF interacts with bone morphologic protein 2 and the complex might regulate the differentiation of chondrocytic cells [27]. Further, Nishida et al. recently demonstrated that CTGF regulates the expression of vascular endothelial growth factor in chondrocytic cells under hypoxic conditions [28]. Based on these findings, the authors suggested a role for CTGF as a "signal conductor" in chondrocytes during endochondral ossification [27,28]. Nevertheless, how CTGF suppression by IL-1 and PGE_2 _modifies the pathophysiology of OA remains unclear. Considering that CTGF might regulate extracellular matrix remodeling and is a pro-fibrotic factor [3], it is possible that CTGF is a downstream factor to inflammatory mediators such as IL-1 and PGE_2_, which modulate the balance between synthesis and degradation of the matrix in the cartilage in inflammatory arthritis.

As for the different bands of CTGF detected in Western blot (Figure 4), this finding may indicate the presence of degradation products and/or posttranscriptional modification by, for example, glycosylation of CTGF expressed in chondrocytes. The existence of similar fragments/byproducts has been reported in previous studies [5]: for example, Yang et al. reported that endogenous human CTGF is a secreted and glycosylated protein of approximately 32 to 38 kDa with 208 kDa of N-linked sugars and a 30-kD core [8]. Yang et al. also demonstrated the presence of differentially degraded forms of CTGF (18 kDa and 24 kDa) in biological fluids, including normal human sera. In addition, Zarrinkalam et al. demonstrated that CTGF protein is expressed in human peritoneal mesothelial cells at 36 to 38 kDa and also at proteolytically processed fragments of lower molecular weight (23 and 25 kDa) [17]. Western blot analysis in the present study revealed predominant bands at approximately 30 to 32 kDa, suggesting that CTGF is deglycosylated in chondrocytes. This point should be further analysed to clarify the cleavage and function of each CTGF isoform in chondrocytes.

In conclusion, we demonstrated that PGE_2 _regulates CTGF expression in chondrocytes at both the mRNA and protein levels via the EP4 receptor. Further investigation of the role of CTGF and the PGE_2 _EP4 receptor in human chondrocytes might provide clues to establish novel therapeutic strategies against osteoarthritis.

## Abbreviations

CTGF/CCN2: Connective tissue growth factor/CCN family member 2; TGF: transforming growth factor, PGE_2_: prostaglandin E_2_; OA: osteoarthritis; PCR: polymerase chain reaction; GAPDH: glyceraldehydes-3-phosphate dehydrogenase; ELISA: enzyme-linked immunosorbent assay; IL-1: interleukin-1

## Competing interests

None of the authors in this study has any financial personal relationship with any organization that could influence (bias) this work.

## Authors' contributions

KM and MM designed and conducted the study. KY, HN, and TK interpreted the data. YS and MB collected the clinical samples and data. All authors approved the final version of the manuscript.
